# Increasing Access to Palliative Care in Cameroon: Progress, Gaps, and Recommendations

**DOI:** 10.3390/nursrep14040263

**Published:** 2024-11-19

**Authors:** Nahyeni Bassah, Anna Santos Salas, Niba Clinton Ambe, Ndzi Eric Ngah

**Affiliations:** 1 Faculty of Nursing, College of Health Sciences, University of Alberta, Third Floor Edmonton, Clinic Health Academy, 11405 87 Avenue, Edmonton, AB T6G 1C9, Canadaniba@ualberta.ca (N.C.A.); 2 Department of Nursing, Faculty of Health Sciences, University of Buea, Buea P.O. Box 63, Cameroon; 3 Palliative Care Unit, Cameroon Baptist Convention Health Services, Tiko P.O. Box 152, Cameroon; ndziericngah@cbchealthservices.org

**Keywords:** palliative care, Cameroon, health care access, practice gaps, nursing

## Abstract

Background/Objectives: Access to palliative care is an urgent global need. Countries with the greatest palliative care needs have limited access. In Cameroon, demand for palliative care is growing due to the rising incidence of life-limiting conditions. Identifying available palliative care services and programs could provide an understanding of access gaps and inform future roadmaps for palliative care development in the country. We aim to map available palliative care services, identify gaps and inform recommendations to promote early access to palliative care in Cameroon. Methods: We undertook a literature review of articles reporting any aspects of palliative care in Cameroon. We searched Embase, MEDLINE, Scopus, PsycINFO, CINAHL, PubMed and gray literature. Data were analyzed thematically using the World Health Organization model for the assessment of palliative care development. Results: We identified 41 articles reporting 21 organizations with some form of palliative care services such as clinical services, education, advocacy and research. These were led mostly by individual health care providers or private and faith-based organizations. Major palliative care initiatives included training in the form of workshops, and adult and pediatric outpatient, in-patient and community-based palliative care. There were few reports of oral morphine production, community engagement, advocacy and palliative care research. Conclusions: Progress in palliative care development was reported in five regions of Cameroon over the last two decades. Findings suggest the need for an intersectoral approach including government, community, and health care stakeholders to achieve sustainable palliative care. This could potentially ensure equitable access to palliative care in Cameroon.

## 1. Introduction

The need for palliative care in Cameroon is on the rise due to the increasing incidence of non-communicable chronic diseases such as cancer, cardiovascular and respiratory diseases [1,2] and the high prevalence of HIV/AIDS in Cameroon [3]. The World Health Organization (WHO) reported an estimate of more than 19,564 new cancer diagnoses and 12,798 cancer deaths in 2022 in Cameroon [4]. These numbers are likely underestimated, given the absence of a national cancer registry in the country. Underestimation of cancer cases has been reported as a major issue in sub-Saharan African countries [5,6]. Moreover, cancer incidence in Cameroon is projected to rise to 27,726 cases by 2035 if cancer control measures are limited [7]. Although the overall adult HIV prevalence has continually decreased since 2004, Cameroon is reported as having the highest HIV infection rates in West Central Africa [3]. In 2022 there was an estimate of 480,232 people living with HIV in a population of about 27,874,766 people, with 9905 new cases recorded that year [8]. Thus, palliative care is needed in Cameroon to prevent and relieve suffering for these patients and their families [9]. People with a life-limiting illness often experience severe pain, fatigue, and anxiety, which has a negative impact on their quality of life [10,11]. This suffering can be relieved with palliative care through early identification, assessment, and treatment of pain and other physical, psychosocial, or spiritual problems [9,11]. Yet, availability of palliative care is limited in over half of the world [12], particularly in low- and middle-income countries (LMICs) [13,14,15]. Only about 14% of the global population who need palliative care are able to access it, with most living in high-income countries [9,16]. Availability and accessibility of palliative care and pain relief is a basic human right for everyone [17,18]. Unfortunately, access to palliative care in LMICs is limited or, too often, completely absent [13,19].

Reports suggest that palliative care was introduced in Cameroon in 2003 through a partnership between Hospice Africa Uganda and the Cameroon Baptist Convention Health Services, a non-profit, faith-based health care organization in the northwest region of Cameroon [20]. This partnership resulted in several initial steps towards palliative care development in Cameroon such as: the opening of a first palliative care unit at the Baptist hospital in Mbingo, Banso [21], the training of the first Cameroonian nurse, Mr Ndikintum Goerge Mbeng in palliative care [22], and the training of seven Cameroonian health care providers in palliative care in 2012 [20,23,24]. Thus, palliative care has existed in Cameroon for over two decades yet to our knowledge no mapping of palliative care developments in the country over these years has been reported. Palliative care development mapping has been undertaken at global [25], regional [22,26], and country levels [27,28] to guide palliative care strategic plans and policies. The most recent global mapping of palliative care developments classified Cameroon under countries with isolated palliative care provision [25]. However, this classification provides limited information about available palliative care services in Cameroon. A synopsis of existing palliative care initiatives in Cameroon can serve to identify gaps, inform future palliative care directions, and recommendations to promote early access to palliative care in Cameroon [29,30]. This mapping can also serve as a resource for patients and families to identify ways in which they could gain access to palliative care services.

## 2. Materials and Methods

We undertook a review of the literature for published articles, reports, books, conference abstracts, and documents reporting any aspects of palliative care in Cameroon. We followed the PRISMA 2020 guidelines for systematic reviews to undertake this review [31]. We searched the following databases: Embase, MEDLINE, Scopus, PsycINFO, CINAHL, PubMed, and grey literature. Sources reporting any aspect of palliative care in Cameroon were included. Our analysis was based on the parameters identified by the WHO model for the assessment of palliative care development including: availability of health policies, use of essential medicines, provision of palliative care (integrated health services), education and training, research, and the empowerment of people and communities [32].

## 3. Results

### Overview of Palliative Care Services in Cameroon

**Figure 1:**
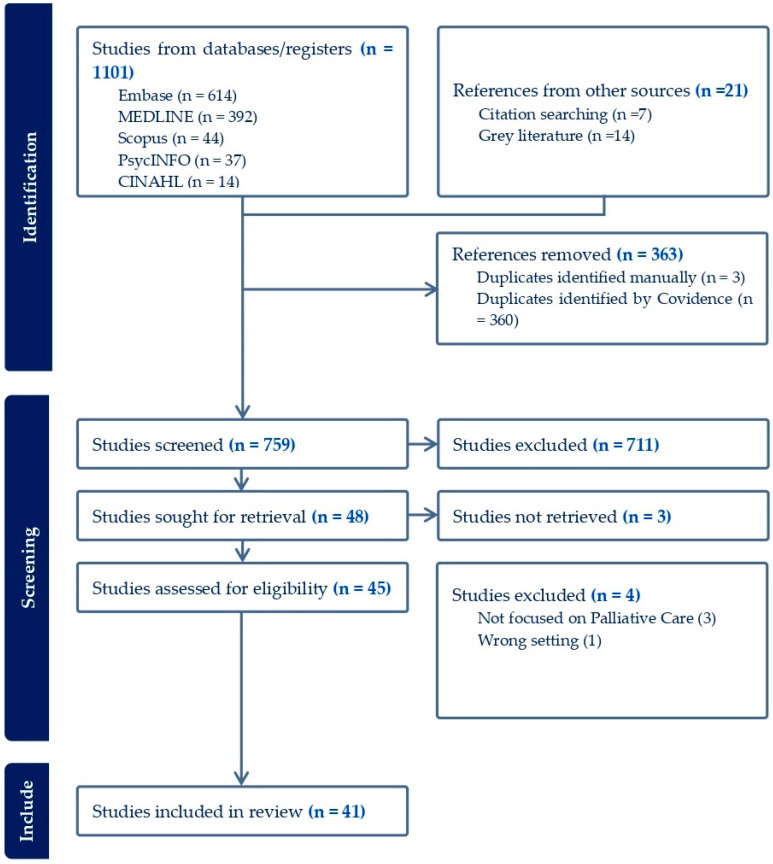
PRISMA Diagram.

A total of 1101 articles were identified from the database searches and 363 were duplicates. We reviewed 738 titles, and 711 were irrelevant. The full text of 27 articles were reviewed and 20 met the inclusion criteria. We obtained an additional seven articles from the reference lists of included articles. A search of the grey literature yielded 14 articles mostly published on palliative and health care organization websites (Figure 1).

A total of 41 published sources between 2006 and 2024 were included to inform this review. They consisted of a wide range of peer reviewed articles, articles on webpages, conference abstracts, and a book chapter. They reported 21 organizations with some form of palliative care programs or initiatives in Cameroon, particularly in the areas of education, advocacy, and clinical care [20,33,34] (Table 1).

The first palliative care unit in Cameroon was opened in 2006 [21], 12 programs started between 2008 and 2013, 7 began between 2014 and 2019, and it is unclear when 1 started (Table 1). These services are concentrated in 5 of the 10 regions in Cameroon, with a majority in the northwest and center regions. In total, 10 programs are run by faith-based organizations, 6 by non-governmental organizations, and 5 are held in government health care facilities. The Cameroon Baptist Convention Health Services is the leading organization for palliative care provision in Cameroon and has served as a mentor for the development of other services [21,22,26]. Most of the palliative care programs in Cameroon have been initiated and led by nurses who either have a diploma (3 years training) or a Bachelor’s degree (4 years training) [22,26]. Others have reported program initiation by nursing aides (1 year training) who have received basic or diploma-level training in palliative care [24,35,36,37]. The Bafoussam regional hospital palliative care program in the west region, for example, was initiated by a nurse aide trained in palliative care and psychosocial counseling [35], while the Banso Baptist hospital integrated palliative care unit was initiated by a Baccalaureate-prepared nurse with a diploma in palliative care [22,38]. Some of these programs have developed progressively to include a multidisciplinary team consisting of nurses, physicians, social workers, chaplains, and pharmacies [24,39]. There was a report of a volunteer international worker supporting and mentoring local teams [40]. The Hospice and Palliative Care Association of Cameroon created in 2009 with headquarters in Bamenda is the main body spearheading advocacy and palliative care education in Cameroon and collaboration with national and international associations in Africa and globally. Table 1 provides a summary of identified palliative care services in Cameroon.

A. Availability of health policies relating to palliative care

Few reports were identified reporting policy development in Cameroon [22]. Some advocacy efforts have been undertaken with local hospital authorities to facilitate integration of palliative care into existing health care services [35]. The Hospice and Palliative Care Association of Cameroon has held conferences to raise stakeholder awareness about palliative care [20,22].

B. Use of essential medicines

Palliative care medications are scarce in Cameroon and morphine is rarely available [20,38,40,41]. The first production of oral morphine in Cameroon is reported in 2006 by the Cameroon Baptist Convention Health Services [20]. The Cameroon Baptist Convention Health Services have a dedicated laboratory that produces liquid oral morphine and distributes to all its health centers [20]. The General Hospital Pharmacy located in Douala in the Littoral region is reported to have started the production of oral morphine in 2014, and it supplies other hospitals in this region. This was facilitated by advocacy efforts by the Volunteers for Palliative Care Association in Cameroon (VOPACA) in collaboration with the American Pain Society who advocated for the acquisition of morphine powder for reconstitution at the General Hospital Douala for nation-wide use [20,42]. Morphine is available for a fee. Collaborative efforts by local institutions to facilitate purchase of morphine at a low cost were reported [40,43]. Nurses with training in palliative care who work at the Cameroon Baptist Convention Health Services are allowed to prescribe oral morphine [22]. Other palliative care medicines available in Cameroon are oxycodone and transdermal fentanyl, dexamethasone, metoclopramide, and diazepam [41].

C. Provision of palliative care in integrated health service delivery platform

Palliative care is not integrated into the national health care system in Cameroon and, thus, its provision is limited, serving few communities [22,26]. Most palliative care services are found in secondary level hospitals: Baptist Hospital Mutengene [44,45], Bamenda Regional Hospital [22,37], Bafoussam Regional Hospital [24,35,46], and Nkongsamba Regional Hospital [20], with one at a primary health care facility (Bonassama District Hospital) [20] and another at a community clinic (Clinique Médicale Camassistance) [43,47]. Most available services provide a combination of outpatient, inpatient, and home-based services [35,43]. Hospital-based services provide home care as a means to ensure follow-up and continuity of care [20,34]. The Bafoussam Regional Hospital palliative care unit, for example, provides both inpatient and outpatient services and supervises 18 mobile clinics that provide home-based palliative care [35]. These programs serve limited numbers of patients that range from 20 patients per week in the entire West region [35], 122 metastatic breast cancer patients in Yaoundé [43] and an average of 10 monthly home visits by the Bonassama District Hospital palliative care program [20].

The components of palliative care that are reported by these programs include: basic symptom control, pain control, psychosocial support, education and counseling, spiritual care, and bereavement support [24,43]. One program reported the role of the family in providing psychosocial support for patients [40]. To facilitate access to services for remote patients and those with low socioeconomic status, some programs provide follow-up care via phone calls, sharing palliative care information through messaging apps and calls and arranging or providing transportation services [24,43].

Several barriers to palliative care provision in Cameroon were reported by programs including the lack of palliative care trained personnel, poor accessibility to palliative care services due to poor roads and transport networks, limited availability of opioids including morphine, lack of government support for palliative care, burnout and psychosocial distress for staff from increased workload, and conflicting schedules between regular hospital work and palliative care visits [20,46]. Interruptions in the provision of both home and hospital-based palliative care services were reported in the northwest and southwest regions of Cameroon due to the armed conflict in this region that started in 2016 [48].

There are incipient initiatives for children’s palliative care in Cameroon [49]. We found reports of a culinary activity for children in end-of-life situations to support the psychosocial and emotional needs of children and their families [50]; a pediatric palliative home care project [39] and a pediatric palliative care outreach program for children with cancer [51]. The pediatric palliative care outreach program for children with cancer is primarily for children with Burkitt’s lymphoma and offers both hospital and home care services [52]. It is led by a nurse aide working in collaboration with the adult palliative care team. They provide biweekly assessment, diagnosis, prescription, and administration of medications and psychosocial and spiritual support. They have a bike and a car to facilitate home care as well as a phone service for patients’ families to call for patient care issues [37].

D. Education and training

There are a few specialist palliative care nurses in Cameroon trained in Hospice Africa Uganda [24,38,53]. The first palliative care training in Cameroon was the “Palliative Care Initiators” course co-organized in 2012 by Hospice Africa Uganda and the Cameroon Baptist Convention Health Services [20,23,24]. This program trained seven Cameroonian health care providers [20]. A second initiator’s course was held in 2013 and facilitated by former course participants [54]. In 2014, a research study evaluating the impact of a 5-day palliative care training program for undergraduate nursing students at the University of Buea was conducted. This training program included content on the concept of palliative care, the need for palliative care in Cameroon, and the communication, cultural, spiritual and ethical aspects of palliative care [55]. This study reported improved palliative care knowledge and self-perceived competence and confidence in palliative care provision [36,55,56]. Other training initiatives reported include the following: a 3-day palliative care initiator course to train health care professionals from 19 health care facilities in the West Region of Cameroon [35,46]; training of health professionals, social workers, and traditional healers to provide palliative care to women with metastatic breast cancer [47]; and in-service training for staff at a new palliative care service by staff members from an established palliative care unit [24]. The Cameroon Baptist Convention Health Services have organized a series of trainings for the multidisciplinary teams in their hospitals. There is no report of any formal training program for adult palliative care specialists in Cameroon. In 2016, a training program was organized to prepare nurse educators in Cameroon to teach palliative care to undergraduate nursing students [57]. The Baptist School of Public Health at Mutengene started a diploma program in Children’s palliative care in 2017 in collaboration with the Mildmay Institute of Health Sciences in Uganda [58].

Palliative care training programs in Cameroon have been offered in person [23,55], and through distance and online teaching [47] by a multidisciplinary group of local and international experts [23,42]. The number and composition of trainees have ranged from: 7 health care providers in Mutengene [20,23], 33 volunteers in Yaoundé [39], 34 medical doctors, paramedics, community members and representatives of civil society, social workers, traditional healers, and psychologists in Douala [42], 50 undergraduate nursing students in Buea [55], and 156 community health professionals, social workers, and traditional healers in Yaounde [43]. A major challenge has been the lack of palliative care clinical placements for trainees [23,55].

These training programs report positive outcomes such as trainees being able to apply their palliative care learning in the provision of palliative care to patients in the hospitals, with improved patient outcomes [23,36,46,55]. Some trained health care professionals have started palliative care services and have trained other providers in their workplace [35,46,54,55]. Team members’ ability to apply their knowledge in practice has been hindered by the lack of morphine, inability to break bad news due to lack of preparation, and high workload [36,46].

E. Research

Published palliative care research in Cameroon is limited. There were some articles on palliative care education, practice development and pediatric palliative care [36,37,40,47,49,51,55,56,59], and conference abstracts on practice development [35,46,47,60,61] published in peer-reviewed journals. There are also a good number of programs that have been published on websites as ‘success stories’ of palliative care education and practice development in Cameroon [24,42,43,57]. There were three reports [21,22,32] and a book chapter on palliative care for cancer patients in Cameroon [20]. These publications report studies that include both qualitative and quantitative methodologies. We did not find any research group that identified as palliative care focused. Most Cameroonian palliative care research initiatives were either self-funded or funded by international donors. The lack of funding is reported as a challenge in scaling up palliative care initiatives in Cameroon [21].

F. Empowerment of people and communities.

The Cameroon Baptist Convention Health Services and Santos Domingo report training of local community volunteers to provide palliative care [33]. The Cameroon Baptist Convention Health Services’ community volunteer program is said to be operational in five regions of the country. This program trains community members to offer services such as comfort care, psychosocial support and spiritual care to patients who are too ill to travel or those in rural and remote communities. They provide daily outreach services and are under the supervision of the hospital palliative care team who joins them on a monthly basis for outreach. These volunteers also help to raise awareness about palliative care as well as help navigate patients for specialist palliative care [44].

## 4. Discussion

Our findings show significant progress in the development of palliative care in 5 of the 10 regions of Cameroon over the last two decades. Palliative care services have been integrated into government, faith-based, and privately owned health care services. There have been multiple palliative care training initiatives for nursing students, health care providers, and volunteers. These have influenced the preparation of palliative care initiators in Cameroon. Emerging areas identified in this review are initiatives designed to advance palliative care policy, access to essential medicines, palliative care research, palliative care leadership, and stakeholder engagement. The Cameroon Baptist Convention Health Board has been a leading organization for palliative care growth in Cameroon. These findings resonate with previous reports that classify Cameroon among countries with isolated palliative care development [25] and raise the need for interventions to improve access to palliative care across the country.

The continued establishment of palliative care programs and services, for both adult and pediatric populations in Cameroon is a critical step. However, in the absence of government policies that support integration, funding, and capacity building for palliative care in the entire health care system in Cameroon, the sustainability of existing services and programs could be compromised. Most services reported served a limited number of patients, averaging 10 to 122 per month. This suggests that existing services may not be readily available to the population they seek to serve. Health care services are considered accessible when they are available, acceptable, affordable and accommodating [62]. In this light, palliative care pioneers in Cameroon could engage with stakeholders, including direct contact with patients and communities to understand their accessibility challenges and jointly determine ways of improving access to palliative care. This collaborative approach is in line with the WHO Public Health Strategy for palliative care [63,64], which recommends community involvement through collective social action [64]. This strategy recommends development of appropriate policies; ensuring adequate drug availability; providing education of policy makers, health care workers, and the public; and implementing palliative care services at all levels throughout the society. A context specific roadmap for palliative care informed by this strategy can serve to improve access and reduce inequities in access to palliative care in Cameroon.

Although most services reported having access to oral morphine (Table 1), it is unlikely that this access is reliable and consistent. Included sources consistently reported limited availability of palliative care medicines in the country. Moreover, only two services reported production of oral morphine available for a fee. In most African countries, access to essential medicines is limited due to financial constraints, high cost of medications, and inadequate human resources, among others [65]. This is likely the case of patients with life-limiting conditions in Cameroon where the majority experience socioeconomic inequities [66]. Sustained advocacy efforts are needed to address opioid restrictive laws and improve accessibility to essential drugs for palliative care in Cameroon. Similar efforts in Rwanda prompted the government to amend its drug regulations to improve access to opioid analgesics. In Tanzania, budget was allocated for the procurement and distribution of morphine and there was training of health care providers on the use of opioid analgesics [28]. Lessons learned in these and other African countries [28] could inform opioid policy for pain management in Cameroon.

Most palliative care services in Cameroon were initiated by nurses with basic to specialist training in palliative care. Nurse-led palliative care models have the potential to increase access to palliative care in resource-limited settings [67]. Thus, developing in-service training programs for health care providers and integrating palliative care content into the undergraduate training curriculum for both health and social care professionals will help develop the generalist workforce for palliative care in Cameroon. Graduate clinical and research specialization programs in palliative care are needed to train Cameroon’s palliative care experts and leaders. These programs could build on existing training programs already piloted and evaluated in the country.

This review showed significant gaps in palliative care leadership, research, and policy in Cameroon. Palliative care research is needed to inform best practices, policy development, and education [68,69]. However, this is subject to the availability of palliative care researchers and research funding. Research training in the form of short courses and fellowships could contribute significantly to advancing research in palliative care in Cameroon.

Volunteers are an integral part of palliative care systems and can be vital in reducing health care system burden, especially in the Cameroonian context where there are limited palliative care resources [70,71]. Volunteers provide services such as bereavement support, emotional support, fundraising, cooking, and family support in hospices, hospitals, and patients’ homes [70,72]. The effectiveness of service provision by volunteers is dependent on continuous training and support [73]. Moreover, engaging communities as partners in palliative care program development is vital to ensuring that programs speak to the unique identities and needs of the peoples and are accessible and effective [71,74].

**Policy recommendations for Palliative Care Development in Cameroon**

Ensuring equitable access to palliative care for all patients and their families living with a life-limiting condition in Cameroon require supportive policies. In this light and considering the above findings and discussion, we have identified the following palliative care policy priority areas. We urge the Cameroon government to develop policies in the following areas:

1. **Establish a national palliative care program at the ministry of public health and health care facilities in Cameroon**: mandate these departments to ensure consistent and equitable access to high-quality palliative care for all Cameroonians regardless of age, setting, socioeconomic status, or religious affiliations among other factors.
2. **Develop a palliative care national framework that supports improved access to palliative care in Cameroon**: establish consultations and collaborations with local and international palliative care experts; learn from existing palliative care frameworks, strategies, and best practices.
3. **Publicly fund palliative care**: include palliative care among list of programs with no fee for service; establish palliative care services in hospitals, establish community/home based and mobile care teams, provide support services for family caregivers and volunteers; provide support services for health care providers involved in palliative care provision.
4. **Ensure consistent availability and affordability of palliative care drugs including morphine**: establish an essential drug list for palliative care in Cameroon; facilitate local production of morphine; train health care providers on effective use of opioid medications.
5. **Integrate palliative care education in health professions curricula and palliative care postgraduate programs**: make palliative care education mandatory for all health and social care professionals at the undergraduate level; develop and make accessible postgraduate programs and continuing professional development opportunities in palliative care for all health and social care professionals in Cameroon.
6. **Increase public awareness about palliative care**: develop awareness programs and campaigns; disseminate information about palliative care through community organizations, health care facilities, health professions training institutions, and social media platforms.
7. **Adopt technology for palliative care delivery**: Use telemedicine and other available technologies to ease access to palliative care, especially for patients and families in rural and remote areas. Use technologies for patient education, symptom tracking and remote monitoring.
8. **Fund palliative care research and knowledge dissemination activities**: provide funding for palliative care researchers; support establishment of palliative care research groups and institutes; provide palliative care research fellowships; support palliative care conferences and workshops.

### Limitations

The number of palliative care services reported in this review are those that were found in the published literature. Other initiatives may have been left out. We undertook a comprehensive search to identify as many palliative care initiatives as possible. The number and types of services offered by published palliative care programs remain unclear, and there was limited information of program approaches, components, and outcomes. We engaged a palliative care leader in this review (NEN) to increase accuracy and comprehensiveness. Stakeholder engagement through research and quality improvement projects can serve to identify knowledge that otherwise may not be found.

## 5. Conclusions

Our review suggests that significant steps have been taken towards advancing palliative care in Cameroon. There is a need for targeted and sustainable strategies to increase stakeholder awareness and involvement in the development of sustainable nation-wide palliative care programs. This can in the long term serve to promote early and equitable access to palliative care in Cameroon.

## Figures and Tables

**Table 1:** Summary of palliative care initiatives in Cameroon.

SN	Name/Institution	Date Established	Region/Location	Focus Area	Program Leadership	Reports Morphine Availability
1	Banso Baptist Hospital Integrated Palliative Care Unit	2006	Northwest, Banso, Kumbo	Hospital and home-based palliative care service provision	Nurse-led	Yes
2	Bamenda Regional Hospital	2008	Northwest, Bamenda	Hospital and home-based palliative care service provision	Nurse-led	Yes
3	Protestant Hospital Ngaoundere Palliative Care Unit	2009	Adamawa, Ngaoundere	Hospital and home-based palliative care service provision	Nurse-led	No
4	Association Santo Domingo-SEG Cameroon, Yaounde	2009	Center, Yaoundé	Advocacy	Possibly nurse-led	No
5	Baptist Hospital Mutengene—Palliative Care Service	2010	Southwest Mutengene	Hospital and home-based palliative care service provision	Nurse-led	Yes
6	Mbingo Baptist Hospital Palliative Care Unit Bamenda	2010	Northwest, Bamenda	Hospital based palliative care service provision	Nurse-led	Yes
7	Council of Heirs International Missions, Yaounde	2010	Center, Yaoundé	Hospital and home-based palliative care service provision	Not clear	Not clear
8	Hospice and Palliative Care Association of Cameroon	2010	Northwest, Bamenda	Advocacy and Education	Not clear	Not applicable
9	St Mary’s Soledad Bamenda	2011	Northwest, Bamenda	Hospital and home-based palliative care service provision	Nurse-led	Yes
10	Etoug-Ebe Baptist Health Centre	2012	Center, Yaoundé	Hospital and home-based palliative care service provision	Physician-led	Yes
11	Integrated Development Foundation Bamenda	2012	Northwest, Bamenda	Hospital based palliative care service provision	Physician-led	No
12	Centre de Sante Catholique de Bikop; Mbalmayo	2013	Center, Mbalmayo	Hospital based palliative care service provision	Not clear	Yes
13	Bafoussam Regional Hospital palliative care program	2013	West, Bafoussam	Hospital and home-based palliative care service provision. Education	Not clear: team consists of a doctor, a pharmacy technician and a nurse aide	Yes
14	Saint Raphael Unit of Palliative Care. Dominican Hospital Saint Martin Porres Yaoundé	2014	Center, Yaoundé	Hospital and home-based palliative care service provision	Not clear	
15	Palliative Care Services, Mboppi Baptist Hospital, Douala	2014	Littoral, Douala	Hospital and home-based palliative care service provision	Physician-led	Yes
16	University of Buea	2014	Southwest	Education	Nurse-led	Not applicable
17	Uphealth Foundation	Possible 2015	Southwest Mutengene	Possibly cancer care	Not clear	Not clear
18	Clinique Médicale Camassistance	2017	Yaoundé	Hospital and home-based palliative care service provision. Education	Physician-led	Yes
19	Bonassama District Hospital Palliative Care program	Possibly 2018	Littoral, Douala	Hospital and home-based palliative care service provision	Physician-led	Yes
20	Nkongsamba Regional Hospital palliative care program	Possibly 2018	Littoral, Nkongsamba	Hospital and home-based palliative care service provision	Physician-led	Yes
21	The Patcha Foundation—Hospice Program, Douala	Not clear	Littoral, Douala	Advocacy	Not clear	Not applicable

## Data Availability

No new data were created for this study.

## Public Involvement Statement

No public involvement in any aspect of this research.

## Guidelines and Standards Statement

This manuscript was drafted against the PRISMA 2020 guidelines for systematic reviews [31].

## References

[1] World Health Organization (2020). The Top 10 Causes of Death. https://www.who.int/news-room/fact-sheets/detail/the-top-10-causes-of-death.

[2] World Health Organization (2021). WHO Takes Steps to Address Glaring Shortage of Quality Palliative Care Services. https://www.who.int/news/item/05-10-2021-who-takes-steps-to-address-glaring-shortage-of-quality-palliative-care-services.

[3] Bekolo C.E., Kouanfack C., Ateudjieu J., Bechem E.T., Ndeso S.A., Tendengfor N., Nsagha D.S., Choukem S.P. (2023). The declining trend in HIV prevalence from population-based surveys in Cameroon between 2004 and 2018: Myth or reality in the universal test and treat era?. BMC Public Health.

[4] Ferlay J.E.M., Lam F., Laversanne M., Colombet M., Mery L., Piñeros M., Znaor A., Soerjomataram I., Bray F. (2024). Global Cancer Observatory: Cancer Today: CAMEROON. https://gco.iarc.who.int/media/globocan/factsheets/populations/120-cameroon-fact-sheet.pdf.

[5] Omotoso O., Teibo J.O., Atiba F.A., Oladimeji T., Paimo O.K., Ataya F.S., Batiha G.E., Alexiou A. (2023). Addressing cancer care inequities in sub-Saharan Africa: Current challenges and proposed solutions. Int. J. Equity Health.

[6] Bray F., Parkin D.M. (2022). Cancer in sub-Saharan Africa in 2020: A review of current estimates of the national burden, data gaps, and future needs. Lancet Oncol..

[7] Ministry of Public Health Cameroon (2020). National Strategic Plan for Prevention and Cancer Control.

[8] World Health Organization (2023). Cameroon Making Progress in the Fight Against HIV. https://www.afro.who.int/countries/cameroon/news/cameroon-making-progress-fight-against-hiv.

[9] World Health Organization (2020). Palliative Care Fact Sheet. https://www.who.int/news-room/fact-sheets/detail/palliative-care.

[10] Worldwide Hospice Palliative Care Alliance (2014). Global Atlas of Palliative Care at the End of Life.

[11] Worldwide Palliative Care Alliance (2020). Global Atlas of Palliative Care.

[12] Brant J.M., Silbermann M. (2021). Global Perspectives on Palliative Care for Cancer Patients: Not All Countries Are the Same. Curr Oncol. Rep..

[13] Knaul F.M., Farmer P.E., Krakauer E.L., De Lima L., Bhadelia A., Jiang Kwete X., Arreola-Ornelas H., Gómez-Dantés O., Rodriguez N.M., Alleyne G.A.O. (2018). Alleviating the access abyss in palliative care and pain relief-an imperative of universal health coverage: The Lancet Commission report. Lancet.

[14] World Health Assembly (2014). Sixty-Seventh World Health Assembly: Strengthening of Palliative Care as a Component of Comprehensive Care Throughout the Life Course. http://apps.who.int/gb/ebwha/pdf_files/WHA67/A67_R19-en.pdf.

[15] World Health Organization (2021). Implementing World Health Assembly Resolution on Palliative Care. https://www.who.int/news/item/12-10-2021-implementing-world-health-assembly-resolution-on-palliative-care.

[16] Anderson R.E., Grant L. (2017). What is the value of palliative care provision in low-resource settings?. BMJ Glob. Health.

[17] Brennan F., Lohman D., Gwyther L. (2019). Access to Pain Management as a Human Right. Am. J. Public Health.

[18] Lohman D., Schleifer R., Amon J.J. (2010). Access to pain treatment as a human right. BMC Med. Educ..

[19] Hannon B., Zimmermann C., Knaul F.M., Powell R.A., Mwangi-Powell F.N., Rodin G. (2016). Provision of Palliative Care in Low- and Middle-Income Countries: Overcoming Obstacles for Effective Treatment Delivery. J. Clin. Oncol..

[20] Dina Bell M.E.H., D’Souza C. (2021). Palliative Care for Cancer Patients in Rural Central Africa: Experiences from Cameroon. Palliative Care for Chronic Cancer Patients in the Community.

[21] Cameroon Baptist Convention Health Board (2010). Strategic Plan 2011–2015. https://www.yumpu.com/en/document/view/36632960/cbchb-strategic-plan-2011-to-2015-cameroon-baptist-convention.

[22] Rhee J.Y., Luyirika E., Namisango E., Powewll R.A., Garralda E., Pons J.J., de Lima L., Centeno C. (2017). APCA Atlas of Palliative Care in Africa.

[23] D’Souza C. (2013). Palliative Care Training in Francophone Africa. https://ehospice.com/africa_posts/palliative-care-training-in-francophone-africa/.

[24] D’Souza C., Djoumessi R., Lonlack C. (2014). Palliative Care Growing in Cameroon. https://ehospice.com/africa_posts/palliative-care-growing-in-cameroon.

[25] Clark D., Baur N., Clelland D., Garralda E., López-Fidalgo J., Connor S., Centeno C. (2020). Mapping Levels of Palliative Care Development in 198 Countries: The Situation in 2017. J. Pain Symptom Manag..

[26] Rhee J.Y., Garralda E., Namisango E., Luyirika E., de Lima L., Powell R.A., López-Fidalgo J., Centeno C. (2018). An Analysis of Palliative Care Development in Africa: A Ranking Based on Region-Specific Macroindicators. J. Pain Symptom Manag..

[27] Fraser B.A., Powell R.A., Mwangi-Powell F.N., Namisango E., Hannon B., Zimmermann C., Rodin G. (2018). Palliative Care Development in Africa: Lessons From Uganda and Kenya. J. Glob. Oncol..

[28] Luyirika E., Lohman D., Ali Z., Atieno M., Mahenge A., Mmbando P., Muinga E., Musyoki D., Mwesiga M.D., Namisango E. (2022). Progress Update: Palliative Care Development Between 2017 and 2020 in Five African Countries. J. Pain Symptom Manag..

[29] Kaasa S., Loge J.H., Aapro M., Albreht T., Anderson R., Bruera E., Brunelli C., Caraceni A., Cervantes A., Currow D.C. (2018). Integration of oncology and palliative care: A Lancet Oncology Commission. Lancet Oncol..

[30] Ferrell B., Temel J., Temin A., Balboni T., Basch E., Firn J., Paice J., Peppercorn J., Phillips T., Stovall E. (2017). Integration of palliative care into standard oncology care: American society of clinical oncology clinical practice guideline update. J. Clin. Oncol..

[31] Page M.J., Moher D., Bossuyt P.M., Boutron I., Hoffmann T.C., Mulrow C.D., Shamseer L., Tetzlaff J.M., Akl E.A., Brennan S.E. (2021). PRISMA 2020 explanation and elaboration: Updated guidance and exemplars for reporting systematic reviews. BMJ.

[32] World Health Organization (2012). Assessing the Development of Palliative Care Worldwide: A Set of Actionable Indicators.

[33] International Association of Hospice and Palliative Care (2022). Global Directory of Palliative Care Institutions and Organizations. https://hospicecare.com/global-directory-of-providers-organizations/search/?idcountry=11.

[34] Oussematou D.T. (2015). Palliative Care Situation in Cameroon. https://palliativecareworks.org/pdf/ConferencePapers/2015_ossematou_dameni.pdf.

[35] Djoumessi R., Lonlack C., Kamgain L., Nsah F., Fetse G. (2016). From Strategy to Implementation: A Palliative Care Network Made Possible in the Naïve Region of West Cameroon. J. Pain Symptom Manag..

[36] Bassah N., Cox K., Seymour J. (2018). Preregistration nursing students’ experiences of a palliative care course in a resource-poor setting. Int. J. Palliat. Nurs..

[37] Tamannai M., Kaah J., Mbah G., Ndimba J., D’Souza C., Wharin P., Hesseling P.B. (2015). An evaluation of a palliative care outreach programme for children with Burkitt lymphoma in rural Cameroon. Int. J. Palliat. Nurs..

[38] Wright M., Clark D., Hunt J. (2006). Hospice and Palliative Care in Africa: A Review of Developments and Challenges.

[39] International Association of Hospice and Palliative Care Global Directory of Palliative Care Institutions and Organizations. Association Santo Domingo-SEG Cameroon 2024. https://hospicecare.com/global-directory-of-providers-organizations/listings/details/1965/.

[40] Schoonover K. (2015). A long way from morphine in rural Cameroon. J. Palliat. Med..

[41] Mapoko B.S.E., Frambo A., Saidu Y., Mbassi E.D.B., Atenguena E., Azemafac K., Kobayashi E., Tabola L., Nkeng G., Sango A. (2023). Assessment of barriers to optimal cancer control in adult cancer treatment centres in Cameroon. Ecancermedicalscience.

[42] D’Souza C. (2014). Palliative Care Training Held in Douala, Cameroon. https://ehospice.com/africa_posts/palliative-care-training-held-in-douala-cameroon/.

[43] Union for International Cancer Control (2023). Strengthening Palliative Care Services for Patients with Metastatic Breast Cancer in Cameroon. https://www.uicc.org/case-studies/strengthening-palliative-care-services-patients-metastatic-breast-cancer-cameroon.

[44] University of Kansas (2023). Palliative Care Department Community Volunteer Project. https://ctb.ku.edu/en/palliative-care.

[45] Cameroon Baptist Convention Health Services (2021). Baptist Hospital Mutengene. https://cbchealthservices.org/hospitals/baptist-hospital-mutengene/.

[46] Djoumessi R., Lonlack C., Kamgain L., Nsah F., Fets G. (2016). Integrating Palliative Care into the Health Care Practice: What Facilitators and Barriers in Cameroon?. J. Pain Symptom Manag..

[47] Nkegoum B., Mboumtou L., Nguedia A., Ndjangueli L., Tiaya L., Dongmo A., Christian T. (2018). Palliative Care Initiative for Cameroonian Women with Metastatic Breast Cancer. J. Glob. Oncol..

[48] Moki E.K. (2021). Cameroon Nurses Seek Extra Care for the Terminally Ill. https://www.voanews.com/a/cameroon-nurses-seek-extra-care-for-the-terminally-ill/6264218.html.

[49] Belika L.M., Makak S.P., Ongotsoyi A.H.P. (2017). Cameroonian context of pediatric palliative care: Ethnanalysis of child diseases. Médecine Palliative.

[50] Nguepy Keubo F.R., Chmielewska K., Nguepy Djoka Tchoumbé R. (2018). Culinary arts as reinforcement for paediatric end-of-life suffering. Rev. Infirm..

[51] Afungchwi G.M., Kruger M., Kouya F., Tih P., McCormick P., Pondy-Ongotsoyi A.H., Hesseling P.B. (2021). Two decades of childhood cancer care in Cameroon: 2000-2020. Pediatr. Blood Cancer.

[52] Wharin P.D., Hesseling P.B., Kouya F., Kaah J., Afungchwi G.M. (2019). An Attempt to Deliver Home-Based Palliative Care to Children with Cancer in Sub-Saharan African Villages-Our Cameroon Experience. Palliat. Med. Care Int. J..

[53] Irish Hospice Foundation (2023). Supporting the Development of Future Leaders of African Palliative Care. https://www.linkedin.com/pulse/supporting-development-future-leaders-african.

[54] D’Souza C., Dameni O. (2014). Palliative Care Training Having Lasting Impact in Cameroon. https://ehospice.com/africa_posts/palliative-care-training-having-lasting-impact-in-cameroon/.

[55] Bassah N., Cox K., Seymour J. (2016). A qualitative evaluation of the impact of a palliative care course on preregistration nursing students’ practice in Cameroon. BMC Palliat. Care.

[56] Bassah N., Palle J.N. (2019). Impact of a Palliative Care Course on Pre-Registration Nursing Students’ Palliative Care Knowledge. Cent. Afr. J. Public Health.

[57] Geneva Foundation for Medical Education and Research (2016). A Palliative Care Course for Nurse Educators, University of Buea, Cameroon. https://www.gfmer.ch/gfmervoices/Mbivnjo-Etheldreda-Leinyuy.htm.

[58] Cameroon Baptist Convention Health Board (2021). Baptist School of Public Health (BSPH), Mutengene. https://cbchealthservices.org/regional-training-center/.

[59] Shey D., Keubo F.R.N. (2021). Experience of psychological distress and the level of resilience by adolescent as care giver in palliative care: A qualitative analysis in the context of hospitalisation. Médecine Palliat..

[60] Ekortarh A., Binam F., Enow-Orock G. (2010). Palliative care: More than just pain relief and symptom control—The role of a psychologist in General Hospital of Yaounde Cameroon. Psycho-Oncol..

[61] Nkofon K.J., Hesseling P.B., Wharin P., Francine K., Afungchwi G.M. (2022). Outreach Palliative Care to Children with Cancer at Banso Baptist Hospital (Bbh) and Mbingo Baptist Hospital(Mbh) North West Cameroon: Nine Years (2013–2021) Experience. Pediatr. Blood Cancer.

[62] Levesque J.F., Harris M.F., Russell G. (2013). Patient-centred access to health care: Conceptualising access at the interface of health systems and populations. Int. J. Equity Health.

[63] Mills J. (2022). Community-based participatory research and Public Health Palliative Care. Prog. Palliat. Care.

[64] Stjernswärd J., Foley K., Ferris F. (2007). The public health strategy for palliative care. J. Pain Symptom Manag..

[65] Yenet A., Nibret G., Tegegne B.A. (2023). Challenges to the Availability and Affordability of Essential Medicines in African Countries: A Scoping Review. Clinicoecon. Outcomes Res..

[66] Ntembe A., Tawah R., Faux E. (2021). Redistributive effects of health care out-of-pocket payments in Cameroon. Int. J. Equity Health.

[67] Bassah N., Vaughn L., Santos Salas A. (2023). Nurse-led adult palliative care models in low- and middle-income countries: A scoping review. J. Adv. Nurs..

[68] Chatland L.E., Harvey C., Kelly K., Paradine S., Bhagat M., Hudson B.F. (2023). Research participation in palliative medicine-benefits and barriers for patients and families: Rapid review and thematic synthesis. BMJ Support. Palliat. Care.

[69] van der Steen J.T., Bloomer M.J., Martins Pereira S. (2022). The importance of methodology to palliative care research: A new article type for Palliative Medicine. Palliat. Med..

[70] Coleman H., Sanderson-Thomas A., Walshe C. (2022). The impact on emotional well-being of being a palliative care volunteer: An interpretative phenomenological analysis. Palliat. Med..

[71] Whitfield K., LaBrie M. (2015). OA58 Community capacity development for enhanced hospice palliative care: Exploring the value of community engagement. BMJ Support. Palliat. Care.

[72] Vočanec D., Lončarek K., Banadinović M., Sović S., Džakula A. (2022). A Qualitative Study on the Position and Role of Volunteers in Integrated Care-An Example of Palliative Care in Croatia. Int. J. Environ. Res. Public Health.

[73] Söderhamn U., Flateland S., Fensli M., Skaar R. (2017). To be a trained and supported volunteer in palliative care—A phenomenological study. BMC Palliat. Care.

[74] Vargas-Escobar L.M., Hernández-Rincón E.H., León-Delgado M.X., Muñoz-Medina S.E., Mantilla-Manoslava N., Correa-Morales J.E., Amorocho-Morales J.D., Sánchez-Cárdenas M.A. (2024). Enhancing rural community engagement through palliative care networks: A scoping review. Health Policy.

